# Insights into Factorless Translational Initiation by the tRNA-Like Pseudoknot Domain of a Viral IRES

**DOI:** 10.1371/journal.pone.0051477

**Published:** 2012-12-07

**Authors:** Hilda H. T. Au, Eric Jan

**Affiliations:** Department of Biochemistry and Molecular Biology, University of British Columbia, Vancouver, British Columbia, Canada; Pohang University of Science and Technology, Republic of Korea

## Abstract

The intergenic region internal ribosome entry site (IGR IRES) of the *Dicistroviridae* family adopts an overlapping triple pseudoknot structure to directly recruit the 80S ribosome in the absence of initiation factors. The pseudoknot I (PKI) domain of the IRES mimics a tRNA-like codon:anticodon interaction in the ribosomal P site to direct translation initiation from a non-AUG initiation codon in the A site. In this study, we have performed a comprehensive mutational analysis of this region to delineate the molecular parameters that drive IRES translation. We demonstrate that IRES-mediated translation can initiate at an alternate adjacent and overlapping start site, provided that basepairing interactions within PKI remain intact. Consistent with this, IGR IRES translation tolerates increases in the variable loop region that connects the anticodon- and codon-like elements within the PKI domain, as IRES activity remains relatively robust up to a 4-nucleotide insertion in this region. Finally, elements from an authentic tRNA anticodon stem-loop can functionally supplant corresponding regions within PKI. These results verify the importance of the codon:anticodon interaction of the PKI domain and further define the specific elements within the tRNA-like domain that contribute to optimal initiator Met-tRNA_i_-independent IRES translation.

## Introduction

The initiation of protein synthesis involves two fundamental processes, ribosome recruitment to an mRNA and selection of a translational reading frame. For most eukaryotic mRNAs, through the concerted action of several eukaryotic initiation factors (eIFs), the 40S ribosomal subunit is recruited to the 5′-end of the transcript and subsequently scans for an AUG initiation codon [1]. Upon anticodon:codon recognition and the subsequent release of initiation factors, the 60S subunit joins to form an elongation-competent 80S ribosome whereby the initiator Met-tRNA_i_ is precisely positioned within the P site of the ribosome.

Although the majority of cellular mRNAs initiate translation via a cap-dependent mechanism, a subset of transcripts and viral RNAs can be expressed through non-canonical, cap-independent means using an internal ribosome entry site (IRES) [2], [3], which mediates gene expression under cellular stress or viral infection when initiation factors are compromised. IRESs are *cis-*acting RNA elements that can directly recruit and position the ribosome, usually utilizing only a subset of canonical initiation factors and *trans*-acting factors [3], [4]. Despite great diversity in the types of IRES elements, they are primarily differentiated based on their sequence and *trans-*acting and initiation factor requirements. Moreover, a subset of IRES mechanisms do not involve scanning but instead these IRESs directly recruit and position the 40S subunit near or at the initiation codon. For example, the Hepatitis C virus (HCV) IRES directly binds to 40S subunits and only requires eIF3 and the ternary complex of eIF2-GTP-Met-tRNA_i_ to position the initiation Met-tRNA_i_ at the AUG start codon [5]. Key to this process, a domain of the HCV IRES adopts a pseudoknot structure, consisting of a four-way junction of coaxially stacked helices, to orient the initiation codon properly on the 40S subunit [6], [7]. These studies indicate that in addition to the activities of initiation factors, the inherent RNA structure of the IRES directly contributes to start-codon positioning.

The intergenic region (IGR) IRES of the *Dicistroviridae* family also contains a pseudoknot structure (PKI) to direct ribosome positioning at the initiation codon. However, unlike the HCV IRES, the IGR IRES has the unique ability to recruit the ribosome in the absence of all canonical initiation factors and establishes the translational reading frame by occupying the ribosomal P site such that the initiating non-AUG codon is positioned in the A site [8]–[11]. Eukaryotic elongation factor (eEF) 1A mediates delivery of the first aminoacyl-tRNA, Ala-tRNA^Ala^, to the A site and eEF2 catalyzes the initial pseudo-translocation step which occurs in the absence of peptide bond formation [12], [13]. eEF2 has also been shown to enhance eEF1A-dependent delivery of the first aminoacyl-tRNA to the ribosomal A site [14]. The IGR IRES utilizes the most streamlined mechanism of action for IRESs [4], [10], [11], [15], [16]. The prototypical member of this viral family is the Cricket paralysis virus (CrPV), which has been used extensively as a model for elucidating the mechanism of factorless IRES-mediated translation.

**Figure 1:**
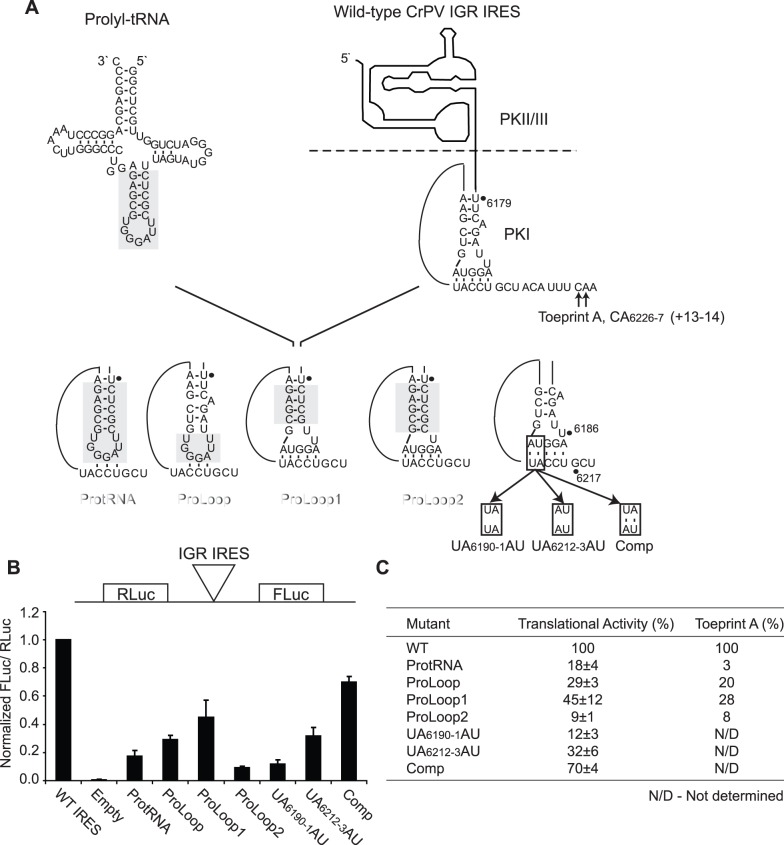
Chimeric IRESs containing prolyl-tRNA and wild-type CrPV PKI regions. (A) *Top.* Schematic of the authentic prolyl-tRNA and wild-type CrPV IRES. The expected location of the positioning toeprint (Toeprint A) is indicated. *Bottom.* Schematic of the dicistronic reporter constructs containing regions of the IRES PKI domain that have been substituted by prolyl-tRNA elements (grey) or bearing mutations in PKI (black box) (B) Translational activities of chimeric and mutant IRESs. *Top.* Uncapped dicistronic reporter RNAs used in translation and toeprinting/primer extension assays. The upstream renilla luciferase (RLuc) and downstream firefly luciferase (FLuc) cistrons are expressed by scanning-mediated and IRES-mediated translation, respectively. *Bottom.* Uncapped dicistronic reporter RNAs containing the wild-type or mutant IGR IRES in the intercistronic space were incubated in RRL in the presence of [^35^S]-methionine. Shown are the average values ± S.D. for the ratios of FLuc to RLuc expression, normalized to the wild-type IGR IRES from at least three independent experiments. (C) Summary of the translational activities and toeprint intensities (from Figure 2) for the chimeric and mutant IRESs. Toeprint intensities were measured as a fraction of the radioactive counts for Toeprint A over the total radioactive counts within each lane, normalized to that of the wild-type IGR IRES.

The secondary and tertiary structures of the IGR IRES are intrinsic to its function. Although there is a lack of conservation at the primary sequence, the secondary structures of the IGR IRESs are well conserved. The IGR IRES adopts an overlapping triple pseudoknot structure (PKI, PKII, and PKIII) which contains two independently folded domains [4], [15], [16]. PKII/PKIII adopts a compact, solvent-inaccessible core which is primarily responsible for ribosome binding [10], [17], [18]. Within this larger domain, stem-loops IV and V have been demonstrated by biochemical studies to mediate critical contacts with the 40S subunit, while the conserved L1.1 region has been predicted to facilitate 80S formation via interaction with the L1 stalk of the 60S subunit [10], [19]–[25]. PKI forms a tRNA-like domain primarily responsible for establishing the translational reading frame [12], [13]. High resolution structural data of the CrPV IGR IRES has demonstrated that PKI mimics an authentic codon:anticodon interaction (Figure 1), where the IRES resembles a P/E hybrid state tRNA [14], [25]. The current paradigm is that this precise tRNA-mRNA mimicry enables the IGR IRES to prime the ribosome into an elongation mode of translation.

**Figure 2:**
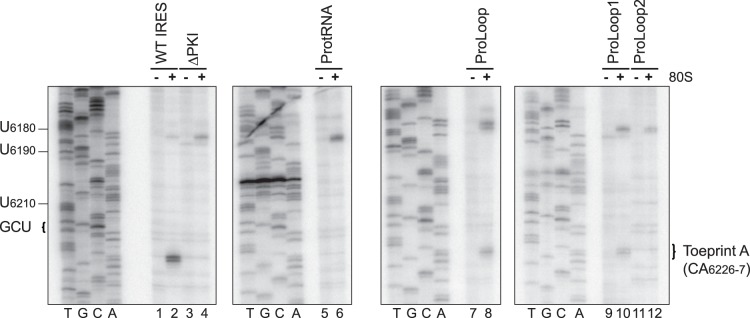
Toeprinting analysis of chimeric IRESs. Dicistronic reporter RNAs containing the wild-type or mutant IGR IRES were incubated in the absence [−] or presence [+] of purified salt-washed HeLa 80S (100 nM). Primer extension analysis was performed using PrEJ69 in the presence of α-[^32^P]-dATP. Reaction products were resolved by denaturing polyacrylamide gel electrophoresis and visualized by autoradiography. The sequencing reactions for each construct are shown on the *left*, with the respective nucleotide numbers as indicated. The locations of the major toeprints are as indicated on the *right*.

Several biochemical approaches have obtained insight into the structure-derived function of the IRES and have demonstrated that the IGR IRES undergoes conformational rearrangements in the progression from the unbound state, to the 40S subunit- and 80S-bound states [10], [17], [21], [22], [25], [26]. In particular, PKI of the IGR IRES may be a structurally dynamic region, as extensive biochemical and enzymatic probing studies have demonstrated that select nucleotides are accessible to reagents that target both single-stranded and helical regions [10], [17]. SHAPE analysis of PKI has also revealed that numerous local conformational changes occur within this region and that specific nucleotides become hypermodified in the 80S- compared to the 40S-bound state [25]. Currently, it is not understood how and at which step these conformational changes contribute to IRES-mediated translation.

A main role of the PKI tRNA mimicry domain is to position the ribosome on the IRES such that translation initiates within a specific reading frame and at a specific non-AUG codon in the ribosomal A site. To gain insight into this process, we performed a comprehensive mutagenesis study targeting three constituents of the PKI tRNA mimicry domain, including the PKI helical stem, the codon:anticodon-like basepairing interactions, and the variable loop region (VLR) and assessed their functional contribution to a key step of IRES-mediated translation initiation – ribosome positioning. Alterations in the helical stem adjoined to the anticodon-like element by introducing nucleotide bulges or basepair deletions tend to reduce IRES translation, suggesting a functional importance of this region to IRES translation. Furthermore, the defect in translation and ribosome positioning observed with the VLR insertion/deletion mutations suggests that there is a stringent requirement in the length of the VLR for IRES activity and that this region may be involved in optimal docking of the IRES within the ribosome to drive translation. Here, we have elucidated the molecular parameters of the PKI tRNA mimicry domain that contribute to IGR IRES-mediated translation and reading frame selection.

## Materials and Methods

### DNA Constructs

The dicistronic luciferase plasmid containing the CrPV IGR IRES has been previously described [10]. Mutant IRES constructs were generated using PCR-based mutagenesis and verified by sequencing. PKI chimeras were designed using the sequence of human prolyl-tRNA [27].

### In vitro Transcription and Translation

The dicistronic DNA construct containing the wild-type or mutant IRES was linearized using XbaI. RNAs were transcribed *in vitro* and subsequently purified using an RNeasy kit (Qiagen). The purity and integrity of the RNAs were confirmed by gel electrophoresis. For *in vitro* translation assays, 0.15 µg of uncapped, pre-folded dicistronic RNAs were incubated in RRL containing potassium acetate to a final concentration of 154 mM. Following incubation for 1 hour at 30°C, the reactions were analyzed by SDS-PAGE and luciferase expression was assessed by [^35^S]-methionine incorporation. The gels were dried and subjected to PhosphorImager analysis (Typhoon, GE).

### Purification of 40S and 60S Ribosomal Subunits

Ribosomal subunits were purified from HeLa cell pellets (National Cell Culture Center) as previously described [10]. HeLa cells were lysed in Triton-X lysis buffer (15 mM Tris-HCl (pH 7.5), 300 mM NaCl, 1% (v/v) Triton X-100, 6 mM MgCl_2_, 1 mg/ml heparin) and the supernatant was subjected to brief centrifugation to remove cellular debris. The supernatant was applied to a 30% (w/w) sucrose cushion containing 0.5M KCl and centrifuged at 100,000 g to pellet ribosomes. Ribosomes were resuspended in buffer B (20 mM Tris-HCl (pH 7.5), 6 mM magnesium acetate, 150 mM KCl, 6.8% (w/w) sucrose, 2 mM DTT) and subsequently treated with puromycin to dissociate ribosomes from the mRNAs. KCl was added to obtain a final concentration of 0.5 M. The dissociated ribosomes were resolved on a 10–30% (w/w) sucrose gradient where the peaks corresponding to the free 40S and 60S subunits were detected by measuring the absorbance at 260 nm. The corresponding fractions were collected, pooled, and concentrated in buffer C (20 mM Tris-HCl (pH 7.5), 0.2 mM EDTA, 10 mM KCl, 1 mM MgCl_2_, 6.8% (w/w) sucrose) using Amicon Ultra spin concentrators (Millipore). The resultant concentrations of the ribosomal subunits were determined by spectrophotometry using the conversions 1 A_260 nm_ = 50 nM and 1 A_260 nm_ = 25 nM for the 40S and 60S subunits, respectively.

### Toeprinting/primer Extension Analysis

Toeprinting analysis of ribosomal complexes in RRL was performed as previously described [11]. 0.4 µg of dicistronic wild-type or mutant IGR IRES RNAs were annealed to primer PrEJ69 5′- GTAAAAGCAATTGTTCCAGGAACCAG - 3′ in 40 mM Tris (pH 7.5) and 0.2 mM EDTA by slow cooling from 65°C to 30°C. Annealed RNAs were added to RRL pre-incubated with either 15 µM edeine or 0.68 mg/mL cycloheximide and containing 20 µM amino acid mix, 8 units of Ribolock (Fermentas) and 154 nM final concentration of potassium acetate (pH 7.5). The reaction was incubated at 30°C for 20 minutes. Following incubation, ribosome positioning was determined by primer extension/reverse transcription using 10 units of AMV reverse transcriptase (Promega) in the presence of 125 µM of each of dTTP, dGTP, dCTP, 25 µM dATP, 0.5 µL of α- [^32^P] dATP (3.33 µM, 3000 Ci/mmol), 8 mM MgOAc, 10 units of Ribolock, 1X buffer E (20 mM Tris-HCl,(pH 7.5), 100 mM KCl, 2.5 mM MgOAc, 0.25 mM spermidine, 2 mM DTT) in the final reaction volume. The reverse transcription reaction was incubated at 30°C for 1 hour, after which it was quenched by the addition of STOP solution (0.45 M NH_4_OAc, 0.1% SDS, 1 mM EDTA). Toeprinting/primer extension analysis using purified 80S was performed in a similar manner using 150 µg dicistronic RNA and 100 nM and 150 nM final concentrations of 40S and 60S, respectively, at 37°C. Following the reverse transcription reaction, the samples were extracted by phenol/chloroform (twice), chloroform alone (once), and ethanol-precipitated. The cDNA was analyzed under denaturing conditions on 6% (w/v) polyacrylamide/8M urea gels, which were dried and subjected to phosphorimager analysis. In each figure, representative gels are shown from at least 2 independent experiments.

## Results

### Regions of PKI can be Substituted by Elements of the prolyl-tRNA Anticodon Stem-loop

Despite some deviations from an authentic codon:anticodon interaction, the striking similarity between the structure of the CrPV PKI and an authentic tRNA-mRNA interaction suggests that the CrPV anticodon-like element may be functionally supplanted by elements of an authentic anticodon stem-loop [25], [28]. To explore this possibility, regions of PKI were swapped with corresponding elements of the prolyl-tRNA anticodon stem-loop and IRES activity was assessed using the standard dicistronic reporter. Prolyl-tRNA was selected as a fitting candidate because a CCU codon encoding proline occupies the ribosomal P site when the CrPV IGR IRES is bound to the ribosome [11]. We reasoned that this approach will help elucidate elements of the CrPV PKI domain distinct from prolyl-tRNA that are important for IRES-mediated translation. To determine if the prolyl-tRNA anticodon stem-loop can functionally replace the anticodon mimic of the IRES, a substitution was made such that the number of base pairs within the helical stem is consistent with the wild-type PKI domain (Figure 1A, ProtRNA). The effect on IRES-mediated translation was assessed in rabbit reticulocyte lysates (RRL) using *in vitro* transcribed dicistronic reporter RNAs containing the wild-type or chimeric IRESs in the intercistronic space (Figure 1B, top). Expression of the upstream renilla luciferase (RLuc) and the downstream firefly luciferase (FLuc) reporters is driven by scanning-mediated and IRES-mediated translation initiation, respectively. For the ProtRNA construct, the substitution was sufficient to confer translational activity albeit weakly, suggesting that sequences of an authentic anticodon stem-loop can impart IRES activity (Figure 1B and C, ProtRNA). To determine if the IRES helical stem or the stem-loop can be independently replaced by the analogous regions of the prolyl-tRNA anticodon stem-loop, chimeric IRES constructs containing substitutions within the PKI domain were generated (Figure 1A). For the ProLoop construct which consists of a composite of the IRES helical stem and prolyl-tRNA stem-loop, 29% of wild-type IRES activity was observed (Figure 1A–C, ProLoop). The reciprocal chimera, the IRES of Proloop2, containing the prolyl-tRNA helical stem and wild-type IRES stem-loop, rendered weaker activity (Figure 1A–C, ProLoop2). Inspection of the secondary structure models of the Type I IGR IRESs reveals that seven of nine members in the subgroup contain a U and G at the base of the stem [15]. Although conserved, crystal structures of the CrPV IRES PKI domain reveal that these bases do not base-pair but are essential for IRES function [25]. To determine if the U and G are important for CrPV IGR IRES-mediated translation, a C to U base substitution was introduced into ProLoop2 to generate ProLoop1 (Figure 1A). Interestingly, this single nucleotide change was sufficient to enhance IRES activity (from 9% to 45% of wild-type) as compared to that of ProLoop2, suggesting that the identity of the U and G at the base of the stem is critical for IRES-mediated translation (Figure 1B and C, ProLoop1). Consistent with the importance of these nucleotides, mutation of U_6185_ to A, C or G inhibited IRES activity [25]. When the same mutation was made in the ProtRNA construct, no significant increase in IRES activity was observed (data not shown), suggesting that the effect of the U and G at the base of the stem is context specific.

The moderate activity exhibited by chimeric IRESs containing the prolyl-tRNA anticodon loop suggests that a three-basepair interaction is sufficient for IRES-mediated translation. To determine if this holds true within the wild-type CrPV IRES, UA_6190-1_ or UA_6212-3_ were individually mutated to their Watson-Crick complements to abrogate the additional basepairs within the tRNA mimicry domain (Figure 1A, UA_6190-1_AU and UA_6212-3_AU). Although both mutations reduced IRES translation, the activities fall within a range that was consistent with the chimeric IRESs containing the ProtRNA anticodon loop (Figure 1B and C). When both mutations were introduced concomitantly to restore basepairing, IRES activity was rescued to ∼70% of wild-type (Figure 1A–C, Comp). These results demonstrate that three basepairs are sufficient to confer IRES translation but that formation of the additional basepairs may further modulate or fine-tune IRES activity, consistent with a previous report also demonstrating that additional PKI basepairing is optimal for *Israeli* acute paralysis virus and Taura syndrome virus IGR IRES activities [29]. Altogether, these results functionally confirm and further reinforce the previous structural and biochemical evidence that the PKI domain mimics an anticodon:codon interaction to drive IRES translation.

IRES-mediated translation involves ribosome recruitment, positioning and the subsequent translocation step. Following recruitment, proper ribosome positioning is directed by formation of an intact PKI which results in the placement of the CCU triplet and GCU initiation codon in the ribosomal P and A sites, respectively [10], [11]. To determine if the defect in IRES-mediated translation for the chimeric IRESs is due to impaired ribosome positioning, toeprinting/primer extension assay was performed using purified salt-washed HeLa 80S. The position of the ribosome on the IRES can be determined by a reverse transcription reaction primed by an oligonucleotide which hybridizes ∼100 nucleotides downstream of the IGR IRES start site. Incubation of the wild-type IGR IRES with purified 80S generated a toeprint at CA_6226-7_ or Toeprint A indicative of proper ribosome positioning (Figure 2, lane 2). The presence of Toeprint A indicates a properly positioned ribosome on the IRES and is generated upon termination of the reverse transcription reaction due to contact made with the leading edge of the ribosome. This stoppage occurs at +13-14 nucleotides downstream of the CCU triplet in the ribosomal P site, given that the first C is designated as the +1 position [10]. A CC_6214-5_GG mutation which effectively disrupted basepairing within PKI and hence ribosome positioning eliminated Toeprint A (Figure 2, lane 4). For the ProtRNA and ProLoop2 chimeras which exhibited weak IRES activity, Toeprint A was not observed, suggesting a deficiency in ribosome positioning (Figure 2, lanes 6 and 12). Both ProLoop and ProLoop1 chimeras generated Toeprint A but at reduced intensities compared to the wild-type IRES, in accordance with their lower translational efficiencies (Figure 1B and C, ProLoop and ProLoop 1, and Figure 2, lanes 8 and 10). These findings suggest that the reduced translational activities of some chimeric IRESs may be due to defective ribosome binding and/or positioning.

### A Single Basepair Deletion is Tolerated in the PKI Helical Stem

**Figure 3:**
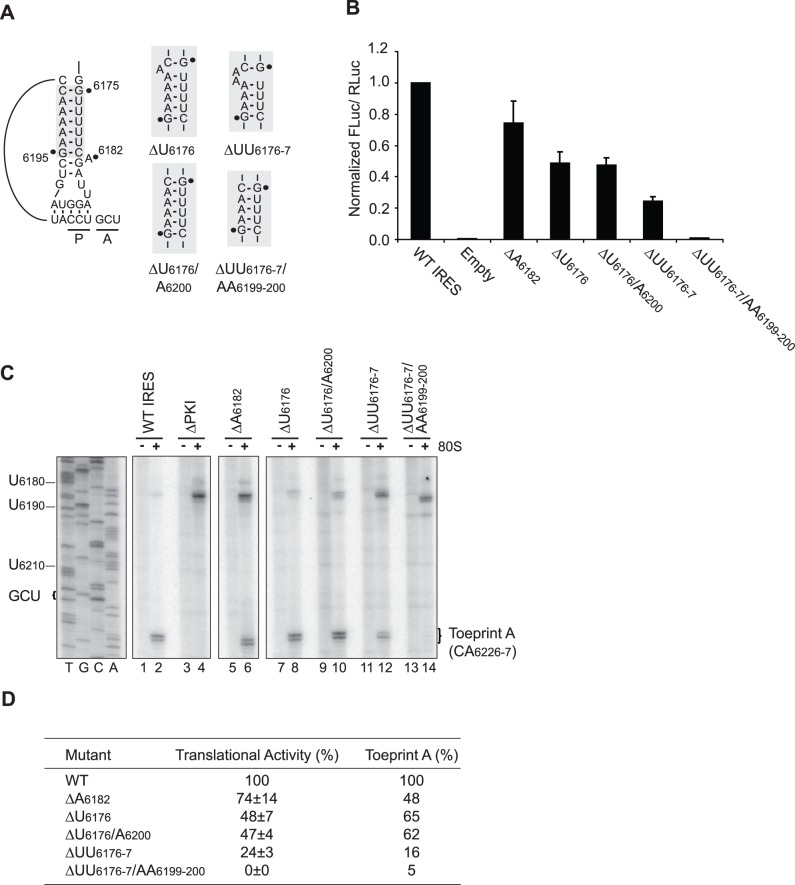
The IRES helical stem tolerates bulges and a 1-basepair deletion. (A) Schematic of the IRES PKI helical stem is shown, with the region bearing mutations highlighted in grey. The respective mutations resulting in a 1-nucleotide bulge (ΔU_6176_), 2-nucleotide bulge (ΔUU_6176-7_), 1-basepair deletion (ΔU_6176_/A_6200_) and 2-basepair deletion (ΔUU_6176-7_/AA_6199-200_) are shown. (B) Translational activities of mutants bearing alterations in the PKI helical stem. Uncapped dicistronic reporter RNAs containing the wild-type or mutant IGR IRES in the intercistronic space were incubated in RRL in the presence of [^35^S]-methionine. Shown are the average values ± S.D. for the ratios of FLuc to RLuc expression, normalized to the wild-type IGR IRES from at least three independent experiments. (C) Toeprinting analysis for IRESs bearing mutations in the helical stem. Dicistronic reporter RNAs containing the wild-type or mutant IGR IRES were incubated in the absence [−] or presence [+] of purified salt-washed HeLa 80S (100 nM). Primer extension analysis was performed using PrEJ69 in the presence of α-[^32^P]-dATP. Reaction products were resolved by denaturing polyacrylamide gel electrophoresis and visualized by autoradiography. The sequencing reaction for the wild-type IRES is shown on the *left*, with the respective nucleotide numbers as indicated. The locations of the major toeprints are as indicated on the *right*. (D) Summary of the translational activities and toeprint intensities for the mutant IRESs. Toeprint intensities were measured as a fraction of the radioactive counts for Toeprint A over the total radioactive counts within each lane, normalized to that of the wild-type IGR IRES.

Having confirmed that the PKI domain can be functionally supplanted by a prolyl-tRNA anticodon loop, we next systematically investigated the three constituents that make up the PKI tRNA mimicry domain. The PKI helical stem of the CrPV IRES resembles that of a tRNA anticodon stem-loop. However, nucleotide bulges are prevalent within the helical stems of IGR IRESs, which do not appear to be conserved from secondary structure predictions [15]. The significance and function, if any, of these bulges have not been investigated. Within the CrPV IRES, A_6182_ protrudes from the helical axis in the ribosome-unbound structure to establish crystal contacts [28]. In the ribosome-bound state, however, this nucleotide extends inward to maintain coaxial stacking within the PKI helical stem [28]. Interestingly, though it is not conserved, deletion of this nucleotide decreased IRES activity to ∼74%, suggesting that this bulge has a minor but functional role in IRES translation (Figure 3A, B and D, ΔA_6182_). To determine if other nucleotide bulges can be tolerated, one or two adjacent nucleotides were deleted from the 5′ proximal region of the helical stem and the resultant effect on IRES activity was assessed (Figure 3A, ΔU_6176_ and ΔUU_6176-7_). Deletion of U_6176_ or UU_6176-7_ reduced IRES translation to ∼50% and 25% of wild-type, respectively (Figure 3B and D). Next, to assess if complete basepairs can be excluded from the helical stem, the complementary nucleotides were also deleted (Figure 3A, ΔU_6176_/A_6200_ and UU_6176-7_/AA_6199-200_). While deletion of one complete basepair rendered the IRES moderately active (∼50%), removal of two complete basepairs was sufficient to abrogate IRES activity (Figure 3B and D, ΔU_6176_/A_6200_ and UU_6176-7_/AA_6199-200_). These results therefore demonstrate that the PKI helical region can tolerate additional bulges, consistent with the lack of conservation in the bulged nucleotides within the helical stem. However, the position of the bulge may be important for optimal IRES activity, as deletion of A_6182_ adversely affected IRES-mediated translation (Figure 3B and D). Additionally, these results also demonstrate that a one-basepair, but not a two-basepair deletion is tolerated in the helical stem, indicating that – and not surprisingly – a minimal helical stem length must be maintained for IRES-mediated translation (Figure 3B and D).

To determine if mutations in the helical stem affected ribosome positioning, toeprinting analysis was performed using purified HeLa 80S in parallel with the wild-type IRES and a mutant deficient in PKI basepairing (Figure 3C). Deletion of A_6182_, U_6176_ or U_6176_/A_6200_ prevented optimal interaction between the codon-like and anticodon-like elements and resulted in reduced Toeprint A intensities (Figure 3C, lanes 5–10 and Figure 3D). Deletion of two adjacent nucleotides in the helical stem, UU_6176-7_, yielded a more severe defect on ribosome positioning, which correlated with the weaker translational activity associated with this mutation (Figure 3C, lanes 11–12 and Figure 3D). Not surprisingly, deletion of two basepairs within the helical stem, UU_6176-7_/AA_6199-200_, resulted in the complete abrogation of Toeprint A in accordance with the lack of translational activity (Figure 3C, lanes 13–14 and Figure 3D). Thus, mutations within the helical stem resulted in a decrease in IRES activity through impairment of pseudoknot formation and ribosome positioning.

### An Optimal Length of the Variable Loop Region (VLR) is Required to Maintain Robust IRES-mediated Translation

**Figure 4:**
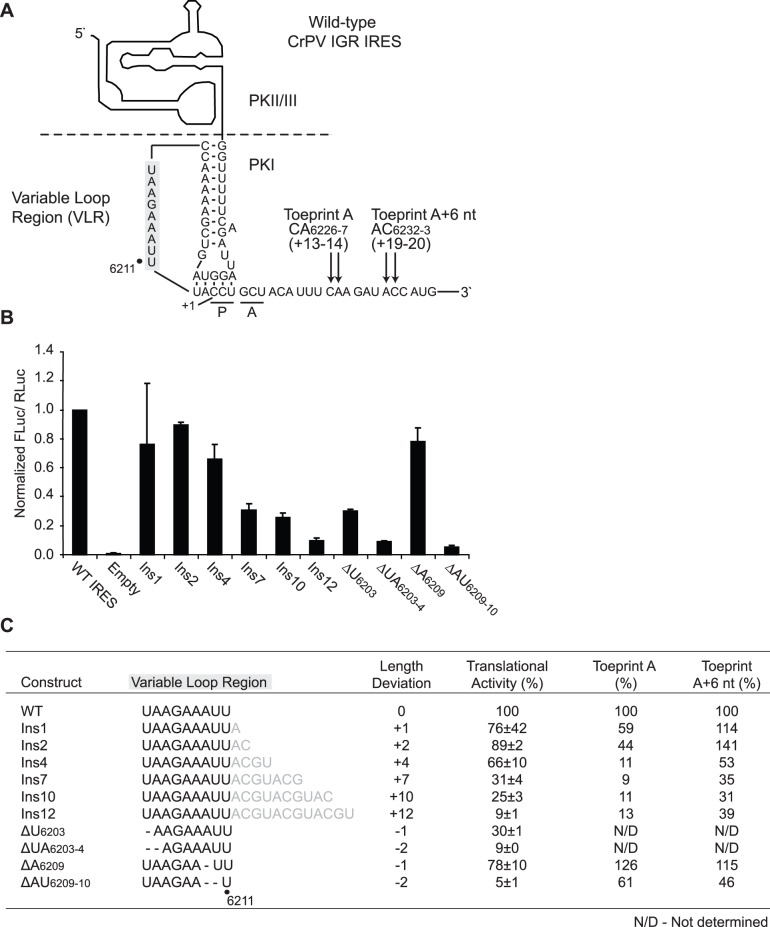
Alterations in the length of the variable loop region (VLR) adversely affect IRES-mediated translation. (A) The schematic of the CrPV IGR IRES secondary structure is shown. The nucleotides constituting the VLR are highlighted in grey. The expected locations of a properly positioned ribosome on the IGR IRES (Toeprint A) and a translocated ribosome (Toeprint A+6 nt) are indicated. The first nucleotide of the codon occupying the ribosomal A site is indicated (+1). **(**B**)** Translational activities of VLR insertion/deletion mutants. Uncapped dicistronic reporter RNAs containing the wild-type or mutant IGR IRES in the intercistronic space were incubated in RRL in the presence of [^35^S]-methionine. Shown are the average values ± S.D. for the ratios of FLuc to RLuc expression, normalized to the wild-type IGR IRES from at least three independent experiments. (C) Summary of IRES translational activities and toeprint intensities in RRL for each construct. Insertion/deletion mutations in the VLR are shown, with inserted and deleted nucleotides highlighted in grey and denoted with dash lines, respectively. Deviations from the wild-type length for each construct are indicated. Toeprint intensities were measured as a fraction of the radioactive counts for Toeprint A or Toeprint A+6 nt over the total radioactive counts within each lane, normalized to that of the wild-type IGR IRES (from Figure 5A).

The codon:anticodon mimicry domain of the IGR IRES is essential in establishing the translational reading frame to initiate translation from the ribosomal A site [8], [11], [25]. A variable loop region (VLR) interconnects the anticodon- and codon-like elements (Figure 4A), and has been previously demonstrated to be susceptible to enzyme and chemical reagents that target single-stranded regions [10], [17]. A comparison of the predicted secondary structure models for the IGR IRESs within the *Dicistroviridae* family reveals that the length of the VLR is not conserved and varies between six to ten nucleotides [15]. Currently, it has not been investigated whether the VLR length contributes to IRES activity. It is possible that the specific length of the VLR of each dicistrovirus IRES may allow for optimal anticodon:codon interaction within PKI. To test this hypothesis, a series of insertion or deletion mutations were generated in this region by either introducing one up to 12 arbitrarily selected nucleotides following U_6211_, the terminal nucleotide in the VLR of the CrPV IGR IRES, or deleting one or two nucleotides (Figure 4A and C). The deletions were made at two distinct locations within the loop to determine whether positional effects are associated with the deletions (Figure 4B and C). Insertion of one, two or four nucleotides within the VLR moderately inhibited IRES activity (66–89% of wild-type IRES activity, Figure 4B and C). However, insertions in excess of four nucleotides exerted a more deleterious effect (9–31% of wild-type IRES activity, Figure 4B and C), with an increasingly more pronounced decrease in IRES translation observed with a greater increase in the length of the loop. Deletion of one nucleotide within the VLR exhibited a positional effect, where the decrease in IRES activity associated with ΔU_6203_ was more drastic than was associated with ΔA_6209_ (30% versus 78%, Figure 4B and C). In both instances, however, a 2-nucleotide deletion exacerbated the inhibitory effects (Figure 4B and C). Thus, these observations indicate that the VLR can tolerate moderate increases but not decreases in length to maintain optimal IRES-mediated translation.

**Figure 5:**
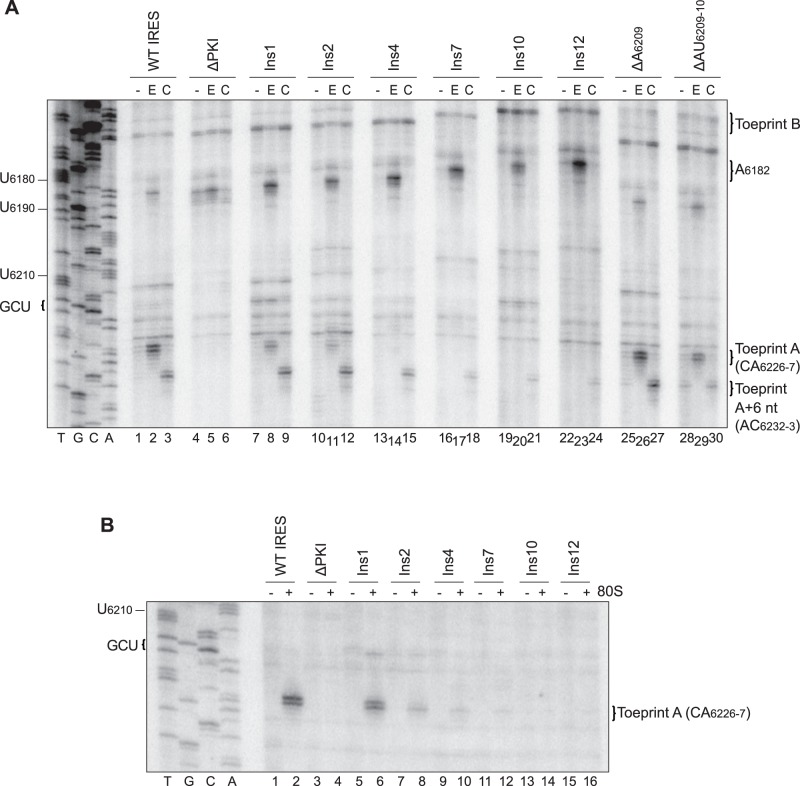
Toeprinting analysis of VLR insertion/deletion mutant IGR IRESs. Dicistronic reporter RNAs containing the wild-type or mutant IGR IRES were incubated in (A) RRL without drug treatment [−], or under treatment with edeine [E] or cycloheximide [C] or (B) in the absence [−] or presence [+] of purified salt-washed HeLa 80S ribosomes (100 nM) as described in the Materials and Methods section. Primer extension analysis was performed using PrEJ69 in the presence of α- [^32^P]-dATP. Reaction products were resolved by denaturing polyacrylamide gel electrophoresis and visualized by autoradiography. The sequencing reactions for the wild-type IRES are shown on the *left*, with the respective nucleotide numbers as indicated. The locations of the major toeprints are as indicated on the *right*. Shown are representative gels from at least two independent experiments.

To determine if repression of IRES-mediated translation for the VLR insertion/deletion mutations is due to impaired ribosome positioning, toeprinting/primer extension assay was performed in RRL. Reactions were performed under the presence of 15 µM edeine, which inhibits delivery of aminoacyl-tRNAs to the ribosomal A site [30], [31], or cycloheximide, which permits two elongation cycles during IRES-mediated translation [11]–[13]. In accordance with previous findings, incubation of the wild-type CrPV IGR IRES with edeine generated a strong toeprint at nucleotides CA_6226-7_, indicative of a properly positioned ribosome on the IRES (Figure 5A, lane 2) [10], [11]. Toeprints were also observed at AA_6161-2_ (Toeprint B) and at A_6182_ (Figure 5A, lane 2), which indicate contacts established between the ribosome and the IRES that effectively impede passage of reverse transcriptase [10], [11]. Upon incubation with cycloheximide, a novel toeprint was observed at AC_6232-3_, 6 nucleotides downstream of Toeprint A, indicating that the ribosome has undergone two translocation cycles (Figure 5A, lane 3, Toeprint A+6 nt). Mutations that abolished the codon:anticodon basepairing within PKI by introducing a CC_6214-5_GG mutation (ΔPKI) eliminated Toeprint A and the translocated toeprint, but not Toeprint B or at A_6182_ (Figure 5A, lanes 4–6). For single and double nucleotide deletions within the variable loop at A_6209_ and AU_6209-10_, treatment with edeine or cycloheximide generated an observable Toeprint A and a translocated toeprint (Figure 5A, lanes 25–30). In general, the decrease in intensities of Toeprint A and the translocated toeprint correlated with the decrease in IRES translational activities, albeit not as closely as that observed with the VLR insertion mutants (Figure 4C and Figure 5A). For the VLR insertion mutations, Toeprint A observed upon edeine treatment decreased in intensity for insertions <4 nucleotides, but was essentially undetectable for insertions of or in excess of 4 nucleotides (Figure 5A, compare lanes 8 and 11 to 14, 17, 20, 23). Due to nucleotide insertions in the VLR, Toeprints B and at A_6182_ migrated slower in the sequencing gel as compared to that of the wild-type IGR IRES (Figure 5A). However and interestingly, cycloheximide treatment yielded a translocated toeprint that was observed for all insertion mutations even in the absence of Toeprint A, albeit with a trend of decreasing intensity that correlated with the observed translational activities (Figure 5A, lanes 9, 12, 15, 18, 21, 24). Thus, for the VLR insertion mutations, the intensity of Toeprint A+6 nt was more correlative with the observed translational efficiencies, as the intensity of Toeprint A approached background levels for constructs which still exhibited IRES activity (Figure 4C, Ins4, Ins7, Ins10, Ins12). To determine if a similar trend is observed with the positioning toeprints using only purified 80S ribosomes, toeprinting analysis was performed with salt-washed, purified HeLa 80S (Figure 5B). A comparable decrease in the intensities of Toeprint A with successive increases in the length of the VLR from 1 to 12 nucleotides was also observed (Figure 5B). Similar to that observed in Figure 5A, the decrease in Toeprint A intensities did not strictly correlate with the IRES translational efficiency (see discussion). In summary, these results show that an optimal length in the VLR is required for proper positioning of the ribosome on the IRES.

### Sequence Identity of the VLR Impacts IRES-mediated Translation

**Figure 6:**
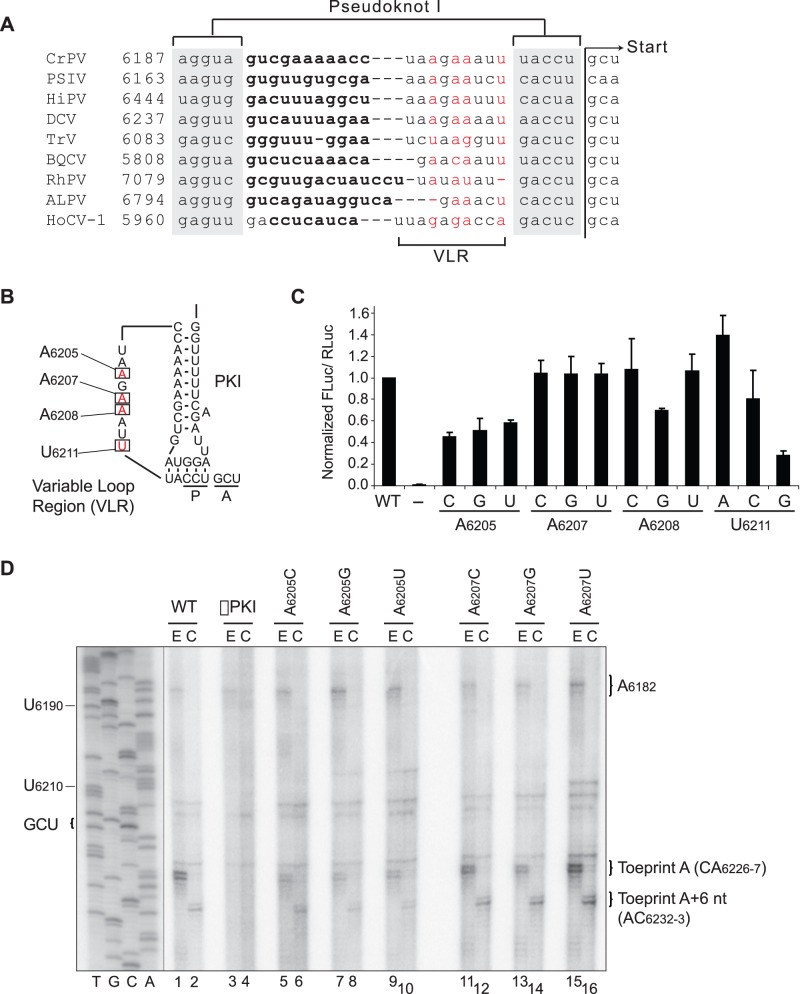
The conserved A_6205_ is important for IRES-mediated translation. (A) Sequence alignment of the variable loop region (VLR) of members of the type I IGR IRESs. Nucleotides involved in basepairing of the helical stem are bolded and those comprising pseudoknot I are highlighted in grey. The nucleotides constituting the VLR are denoted, with conserved positions highlighted in red. The assignments are made based on the secondary structure predictions of the IGR IRESs. The translational start site is indicated. (B) The schematic of the PKI domain is shown with the nucleotides constituting the VLR highlighted in grey. Four conserved nucleotides within the VLR, A_6205_, A_6207_, A_6208_ and U_6211_ are denoted by boxes and were mutated to each of the alternate Watson-Crick bases. (C) Translational activities of VLR point mutants. Uncapped dicistronic reporter RNAs containing the wild-type or mutant IGR IRES in the intercistronic space were incubated in RRL in the presence of [^35^S]-methionine. Shown are the average values ± S.D. for the ratios of FLuc to RLuc expression, normalized to the wild-type IGR IRES from at least three independent experiments. (D) Toeprinting/primer extension assay of VLR point mutants. Dicistronic reporter RNAs containing the wild-type or mutant IGR IRES were incubated in RRL under treatment with edeine [E] or cycloheximide [C] as described in the Materials and Methods section. Primer extension analysis was performed using PrEJ69 in the presence of α- [^32^P]-dATP. Reaction products were resolved by denaturing polyacrylamide gel electrophoresis and visualized by autoradiography. The sequencing reactions for the wild-type IRES are shown on the *left*, with the respective nucleotide numbers as indicated. The locations of the major toeprints are as indicated on the *right*. Shown is a representative gel from at least two independent experiments.

Although the length is not conserved, a sequence alignment of at least six of nine members of the type I IGR IRESs indicates the presence of several conserved nucleotides in the VLR (Figure 6A) [25]. Specifically, nucleotides A_6205_, A_6207_, A_6208_ and U_6211_ of the CrPV IRES appear to be moderately conserved [25] (Figure 6A). To determine if the sequence of the VLR is important for IRES-mediated translation, point mutations were introduced at the conserved bases, A_6205_, A_6207_, A_6208_, and U_6211_ of the CrPV IGR IRES and IRES translational efficiency was assayed using the standard dicistronic reporter system (Figure 6B). Mutation of A_6205_ to each of the alternative Watson-Crick bases exhibited a moderate inhibition of IRES-mediated translation (Figure 6C, ∼50% of wild-type). In contrast, mutation of A_6207_ exerted a negligible effect on IRES activity (Figure 6C). Interestingly, the effect on IRES translation by mutation of A_6208_ and U_6211_ was dependent on nucleotide identity (Figure 6C). Mutating A_6208_ to G and A_6211_ to G moderately inhibited IRES translation whereas U_6211_ to A slightly increased IRES activity by 30% (Figure 6C). Mutating A_6208_ and U_6211_ to other alternative nucleotides did not alter IRES translation (Figure 6C). These results demonstrate that A_6205_ is necessary for optimal IRES translation and that the nucleotide identity at A_6208_ and A_6211_ but not at A_6207_ affects IRES activity.

Because mutation at A_6205_ adversely affected IRES activity in a nucleotide-independent manner, toeprinting/primer extension assay was performed in RRL to determine if mutation at this position conferred general defects in ribosome positioning and/or translocation. As a control, ribosome positioning was monitored on mutant IRESs containing mutations at A_6207_, as mutations at this position did not affect IRES activity (Figure 6C). Under edeine treatment, mutations at A_6205_ yielded a consistent decrease in the intensities of the positioning toeprints, with no significant decrease in the A_6182_ toeprint compared to wild-type (Figure 6D, lanes 5, 7, 9). Similarly, a decrease in the intensities of the translocated toeprints was also noted under cycloheximide treatment, consistent with the observed decrease in translational activities (Figure 6D, lanes 6, 8, 10). For mutations at A_6207_, the intensities of the positioning and translocated toeprints observed under edeine and cycloheximide treatment were similar to wild-type and corresponded with the observed translational activities (Figure 6D, lanes 11–16). Thus, these results suggest that A_6205_ but not A_6207_ has a role in ribosome positioning. It remains to be investigated whether mutations at A_6205_ alter the structure of the PKI domain, which in effect may impair ribosome positioning on the IRES.

### IRES-mediated Translation Initiation is Permissible at an Adjacent and Overlapping Alternate Start Site

In canonical translation initiation, recognition of the translational start site occurs through optimal codon:anticodon interaction involving initiator Met-tRNA^Met^ in the eIF2- and GTP-bound ternary complex [1]. During factorless IGR IRES-mediated translation, the PKI domain of the IGR IRES plays an analogous role by precisely mimicking an authentic codon:anticodon pairing in the ribosomal P site, establishing the translational reading frame and positioning the subsequent codon in the A site such that the message is appropriately decoded [11], [25], [32], [33]. Previous biochemical studies have suggested that interactions within PKI are dynamic and that coordinated conformational changes within PKI are necessary to engage the ribosome into a pretranslocation state [10], [15], [25]. Furthermore, recent data suggest that a subset of IGR IRESs can initiate translation in an alternate reading frame through an IRES-dependent mechanism [34]. These observations suggest that proper reading frame selection for IRES-mediated translation initiation may be intrinsic within the tRNA-like PKI domain. Thus, translation initiation at an alternate start site may be permissible provided that critical basepairing interactions within PKI are maintained.

**Figure 7:**
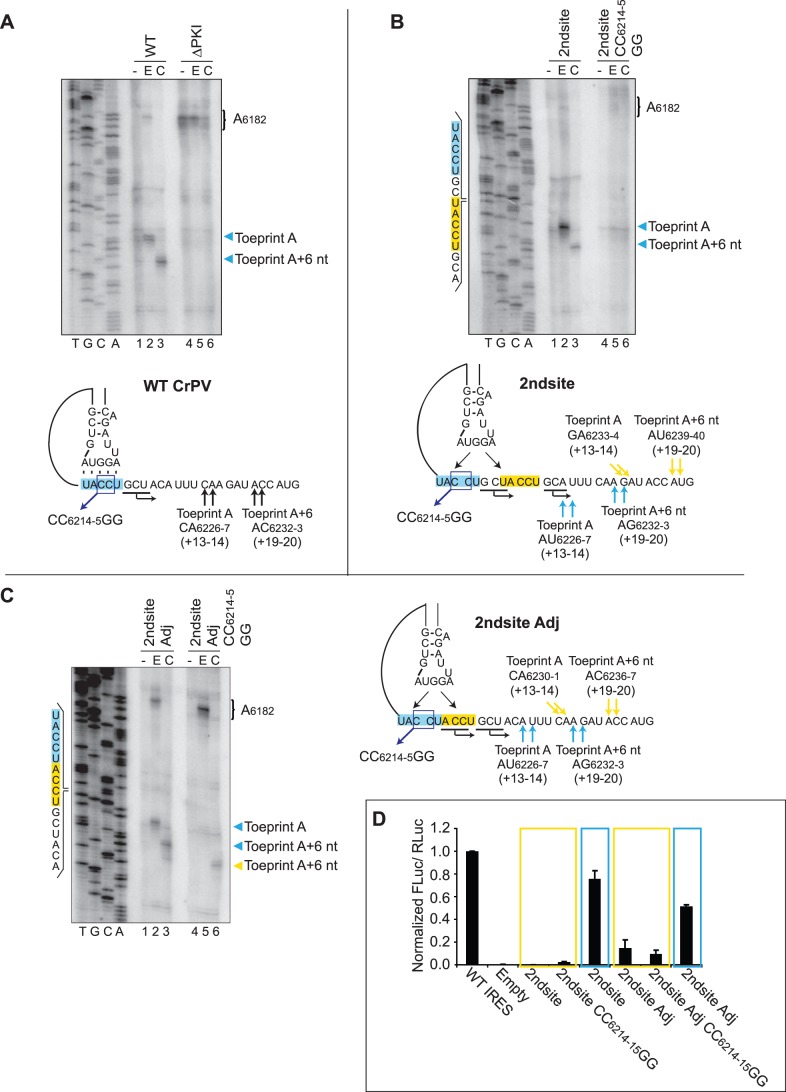
IGR IRES-mediated translation initiation at an alternate translational start site. Schematic of the construct of the wild-type IGR IRES PKI domain (A, *bottom*) and constructs which contain an alternate translational start site 2 nucleotides downstream [2ndsite] (B, *bottom*), or adjacent to and overlapping with the authentic start site [2ndsite Adj] (C, *right*). The authentic and inserted translation start sites are highlighted in blue and yellow, respectively. The expected locations of a properly positioned ribosome (Toeprint A) and a translocated ribosome (Toeprint A+6 nt) from the authentic and inserted sites are denoted by arrows of the corresponding colors for each construct. (A and B (*Top*), C, (*left*)) Toeprinting analysis for the respective constructs. Dicistronic reporter RNAs containing the wild-type or mutant IGR IRES were incubated in RRL without drug treatment [−], or under treatment with edeine [E] or cycloheximide [C] as described in the Materials and Methods section. Primer extension analysis was performed using PrEJ69 in the presence of α- [^32^P] dATP. Reaction products were resolved by denaturing polyacrylamide gel electrophoresis and visualized by autoradiography. The sequencing reactions for the constructs are shown, with the nucleotides corresponding to the authentic and inserted sites highlighted in blue and yellow, respectively. The locations of the major toeprints are as indicated to the right of the gel in the same color. (D) IRES translational activities of reporter constructs containing alternate translational start sites. Uncapped dicistronic reporter RNAs containing the wild-type or mutant IGR IRES in the intercistronic space were incubated in RRL in the presence of [^35^S]-methionine. Shown are the ratios of FLuc to RLuc expression, normalized to the wild-type IGR IRES. Translational activities monitored in the reading frame of the authentic and inserted start sites are highlighted in blue and yellow, respectively. Shown are average values from at least three independent experiments ± S.D.

To test this, UACCU_6212-6_, which constitutes the codon-like element of PKI of the IRES, was duplicated and inserted either two nucleotides downstream of the authentic codon-like element of the IGR IRES, or immediately adjacent to but overlapping by one nucleotide (Figure 7B and C, 2ndsite and 2ndsite Adj, respectively). Because the inserted sequence exhibited base complementarity to the anticodon-like element of the IRES, it provided an alternate site for PKI basepairing. The inserted sequence was introduced into an alternative reading frame; consequently, two distinct reporter constructs were generated to differentiate between selection of the authentic (highlighted blue box) or inserted translational start sites (highlighted yellow box) (Figure 7A–C). Where necessary, the appropriate mutations were made near the reporter start codon such that initiation from the authentic or inserted start sites could be monitored by FLuc expression. Within these dicistronic reporter constructs, IRES-mediated FLuc translational activity was normalized to translation of the upstream RLuc, which served as a normalization control between experiments.

For the construct where the second codon-like element was inserted two nucleotides downstream of the authentic site, negligible reporter expression from the inserted site was observed (Figure 7D, 2ndsite, yellow box), in contrast to robust IRES translation that was observed from the authentic site (Figure 7D, 2ndsite, blue box). To determine if the authentic and inserted sites competed for basepairing with the anticodon region of PKI, a CC_6214-15_GG substitution mutation was introduced (Figure 7B, 2ndsite CC_6214-15_GG), which effectively disrupted basepairing at the authentic site to potentially promote basepairing exclusively at the inserted sequence. Interestingly, the CC_6214-15_GG mutation resulted in negligible FLuc expression (Figure 7D, 2ndsite CC_6214-15_GG, yellow box), suggesting that the authentic translational start site may be preferentially selected in IRES-mediated translation. When the second codon-like element was introduced adjacent to the authentic site, translation from the inserted site was observed (9–14% of the wild-type IGR IRES activity) (Figure 7D, 2ndsite Adj, yellow box). Although within this construct, the authentic site supported only ∼51% of the wild-type IGR IRES activity (Figure 7D, 2ndsite Adj, blue box), the extent of IRES translation was higher compared to the inserted site, further suggesting an inherent preference for the authentic translational start site even when an alternate site is accessible (Figure 7D, 2ndsite Adj, compare yellow and blue boxes). Disrupting the authentic site by introducing the CC_6214-15_GG mutation did not significantly affect translation from the adjacent inserted site (Figure 7D, 2ndsite Adj CC_6214-15_GG). Taken together, these findings indicate that IRES-mediated translation initiation is permissible at an alternate start site, but only when the inserted site is located proximally to the authentic site.

To confirm that ribosomes are in fact positioning at both sites of initiation, toeprinting analysis was performed in RRL (Figure 7A–C). For the 2ndsite construct, treatment with edeine generated a single observable toeprint at nucleotides AU_6226-7_, indicating a properly positioned ribosome at the authentic translational start site (Figure 7B, lane 2). Treatment with cycloheximide generated a translocated toeprint at nucleotides AG_6232-3_, six nucleotides downstream, which was consistent with a ribosome positioned after two cycles of translocation (Figure 7B, lane 3). Provided that ribosomes were positioned at the inserted site of the 2ndsite construct, toeprints should be observed at GA_6233-4_ and AU_6239-40_ under edeine and cycloheximide treatment, respectively (Figure 7B, bottom). However, no detectable toeprints were observed under edeine or cycloheximide treatment at either of these sites even when the authentic site was disrupted (Figure 7B, lanes 5–6), consistent with the observation that IRES-mediated translation initiated exclusively from the authentic codon-like element. For the 2ndsite Adj construct, a single discrete toeprint corresponding to a properly positioned ribosome at the authentic translational start site was observed at nucleotides AU_6226-7,_ which is inconsistent with the observation that both sites supported translation (Figure 7C, lane 2). Only upon treatment with cycloheximide were two distinct toeprints discernible: a higher intensity toeprint at AG_6232-3_ and a lower intensity toeprint at AC_6236-7_ which corresponded to translocation from the authentic and inserted sites, respectively (Figure 7C, lane 3). Similarly, the 2ndsite Adj CC_6214-5_GG construct, which supported translation initiation exclusively from the inserted site, generated an observable toeprint at AC_6236-7_ only upon cycloheximide, but not edeine treatment (Figure 7C, lanes 5–6). In summary, these results indicate that although translation initiation mediated by the CrPV IRES is permissible at an alternate start site, optimal translation initiation occurs at the authentic start site.

## Discussion

The recent acquisition of a high-resolution structure of the CrPV IGR IRES PKI domain has yielded significant insights into the remarkable ability of structured RNAs to manipulate the translational machinery [25], [28]. The current paradigm that the IGR IRES mimics an authentic tRNA-mRNA interaction to direct factorless IRES translation and set the ribosome into a specific reading frame sufficiently accounts for the unique characteristics of this mode of translation. To complement previous structural and biochemical studies, we have used novel mutations to probe the tRNA mimicry domain to identify specific elements that confer IRES activity and the ability to recruit the ribosome and select the translational reading frame. Deviations in the length of the PKI helical stem, VLR, or the anticodon:codon basepairing resulted in reduced IRES translation, suggesting that the tRNA mimicry domain of the wild-type IRES has been optimized for IRES translation. Furthermore, we have identified specific nucleotides within the VLR that contribute to IRES activity. These studies have revealed the structural constraints and parameters of the anticodon mimicry domain for IRES-mediated translation and provide a molecular framework for understanding how the IGR IRES hijacks and engages the ribosome into an elongation-competent mode.

The disordered nature of the VLR in the ribosome-bound state is a conserved feature for both the CrPV and the related *Plautia stali* intestine virus and suggests that there may be functional implications in the flexible nature of this region [28]. Here, we demonstrate that the wild-type CrPV IGR IRES has preserved an ideal length of the VLR for translation (Figures 4 and 7). The reduced translational activities of the VLR insertion mutants and mutants bearing alternate start sites suggest that there are constraints within the VLR and PKI domain that limit the range for basepairing for pseudoknot formation and reading frame selection by the IGR IRES (Figure 4 and 7D). While a robust positioning toeprint was noted for the wild-type IRES, translationally active mutants bearing VLR insertions generated observable toeprints only upon translation elongation (Figure 5A). A similar phenomenon was also observed when IRES translation initiated from an adjacent start site, which effectively increases the length of the VLR (Figure 7C). These results suggest that the extraneous sequences within the VLR – either by nucleotide insertion or selection of the downstream alternate start site – may impede optimal docking of the IRES into the ribosomal P site, possibly due to limited space in the P site. Alternatively but not exclusively, increasing the length of the VLR may promote more transient interactions between the IRES and the ribosome such that the reverse transcription reaction cannot sufficiently capture the positioning toeprint during primer extension. In both scenarios, the translocated toeprint was observed under cycloheximide treatment, suggesting that delivery of the Ala-tRNA^Ala^ to the ribosomal A site and subsequent translocation steps further stabilize the IRES-80S complex (Figure 5A and Figure 7C). Although further experimentation will be required to differentiate between these two models, the uncoupling of the positioning toeprint to the IRES translational activities observed here underscores the need to revisit the previous use of the positioning toeprint as a diagnostic for translational activity [24], [35].

We have recently demonstrated that a subset of IGR IRESs can initiate translation in the +1 reading frame through a non-canonical basepair adjacent to PKI [34]. Here, our data hints at the propensity of the CrPV IGR IRES to initiate translation at a different start site and potentially in an alternate reading frame, as an overlapping and adjacent codon-like element supported IRES-mediated translation (Figure 7D). However, more robust activity was consistently observed from the authentic translational start site in both the translation and toeprinting assays, indicating an optimal context for IRES-mediated translation (Figure 7). Interestingly, the lack of increase in IRES translation from the alternate site when the authentic site was compromised by base substitution suggests little competition between initiation from the two sites (Figure 7D), and that distinct conformations of the IRES establish anticodon:codon basepairing, possibly prior to the IRES setting the ribosome into a reading frame in the P site. The dynamic nature of the tRNA mimicry domain, supported by structural and enzymatic probing data, may enable the IRES to sample for the appropriate translational reading frame through basepairing between the anticodon- and codon-like elements [10], [17], [25]. It will be of interest to further examine the implications of the dynamic nature of the PKI domain on IRES-mediated translation and to determine whether the differences in the VLR lengths of other dicistroviruses are also ideal for IRES translation.

Our results indicate that the nucleotide identity within the VLR has a role in IRES activity. Translation initiation at the adjacent or downstream alternate translational start sites effectively increases the length of the VLR by 4 or 7 nucleotides, respectively (Figure 7B and C). While the data is complementary, the differences in activities of the 2ndsite Adj and 2ndsite mutants with VLR mutants harboring 4- and 7-nucleotide insertions imply that the sequence of the VLR is a critical determinant of IRES activity. In support of this, mutating the conserved A_6205_, and A_6208_ and U_6211_ to specific nucleotides resulted in a decrease in IRES activity (Figure 6C). It remains to be investigated whether A_6205_ or other nucleotides within the VLR affect the conformation of PKI or may interact, possibly transiently, with the ribosome, IRES and/or the incoming tRNA to mediate IRES-dependent translation.

Structural comparison between the anticodon mimic of the IRES and an authentic tRNA anticodon:codon pair reveals remarkable similarities, although some deviations occur in the vicinity of the PKI basepairs [25]. Most notably, the three bases constituting the anticodon in the initiator tRNA have been replaced by five analogous basepairing interactions within PKI, and some structural rearrangements are introduced which are not observed in the authentic tRNA-mRNA interaction [25]. Our results demonstrate that an authentic tRNA anticodon stem-loop can impart translational activity and provide further biochemical evidence in support of the current paradigm (Figure 1). Additionally, abrogation of the additional basepairs within PKI of the CrPV IRES– by mutation of UA_6190-1_ or UA_6212-3_ to their Watson-Crick complements such that the basepairing more closely resembles a true codon:anticodon pairing – suggests that three basepairing interactions can confer IRES translation, albeit weakly (Figure 1). Additional basepairs within PKI likely evolved to further modulate or fine-tune IRES translational efficiency. For instance, deletion of A_6191_ results in an enhancement in IRES translation, suggesting that IRES activity is not maximal but is optimized in the context of the viral genome [25]. Our results also demonstrated the modular nature of the IGR IRES. This observation is not unprecedented, as derived IRESs comprised of the ribosome-binding and tRNA mimicry domains from two distinct IGR IRES classes can mediate IRES translation [35]. The results herein present an enticing possibility that the IRES may have originated from tRNA-like elements or evolved from the anticodon stem-loop of an authentic tRNA, and subsequently acquired mutations that enable it to function optimally for viral IRES-mediated translation.

Viruses have evolved unique mechanisms for translating their genomes as a strategy to hijack the ribosome during infection when cap-dependent translation is compromised. Both HCV and CrPV IGR IRESs have acquired a pseudoknot to directly manipulate the ribosome and position it properly at the initiation codon. However, each IRES utilizes the pseudoknot distinctly. The HCV pseudoknot acts as a connector region to link the ribosome binding domain and the initiation codon-containing domain and orients the HCV initiation codon in the mRNA binding cleft of the 40S subunit to presumably mediate binding of the ternary complex eIF2-GTP-Met-tRNA_i_ [7]. In contrast, the IGR IRES pseudoknot does not require any factors but instead the PKI domain, which connects in *cis* the anticodon mimicry stem-loop with the initiation codon, occupies the ribosomal P site to engage the ribosome to initiate translation from the A site. For both IRESs, the molecular framework of the pseudoknot domains has been optimized for efficient IRES translation [7, this work]. The current study provides the basis for future work into understanding how different regions of the PKI domain, especially specific elements of the VLR, coordinately function and direct IRES-mediated translation. As we gain more structural and biochemical insights into these viral tRNA-like elements, it will be interesting to see whether other viral and cellular IRESs use pseudoknot structures as a common strategy for ribosome positioning and reading frame selection. Finally, it is interesting to note that some plant RNA viruses also utilize a tRNA-like element within their 3'UTR to mediate viral translation and replication, suggesting that tRNA mimicry may be a more general strategy for viral translation [36], [37].

## References

[1] Pestova TV, Lorsch JR, Hellen CU (2007) The Mechanism of Translation Initiation in Eukaryotes. In: Mathews MB, Sonenberg N, Hershey J, editors. Translational Control in Biology and Medicine. Cold Spring Harbor, NY: Cold Spring Harbor Laboratory Press. 87.

[2] Hellen CU, Sarnow P (2001) Internal ribosome entry sites in eukaryotic mRNA molecules. Genes Dev 15: 1593–1612. 11445534 10.1101/gad.891101

[3] Doudna JA, Sarnow P (2007) Translation Initiation by Viral Internal Ribosome Entry Sites. In: Mathews MB, Sonenberg N, Hershey J, editors. Translation Control in Biology and Medicine. Cold Spring Harbor, NY: Cold Spring Harbor Laboratory Press. 129.

[4] Kieft JS (2008) Viral IRES RNA structures and ribosome interactions. Trends Biochem Sci 33: 274–283. 18468443 10.1016/j.tibs.2008.04.007 PMC2706518

[5] Pestova TV, Shatsky IN, Fletcher SP, Jackson RJ, Hellen CU (1998) A prokaryotic-like mode of cytoplasmic eukaryotic ribosome binding to the initiation codon during internal translation initiation of hepatitis C and classical swine fever virus RNAs. Genes Dev 12: 67–83. 9420332 10.1101/gad.12.1.67 PMC316404

[6] Berry KE, Waghray S, Doudna JA (2010) The HCV IRES pseudoknot positions the initiation codon on the 40S ribosomal subunit. RNA 16: 1559–1569. 20584896 10.1261/rna.2197210 PMC2905755

[7] Berry KE, Waghray S, Mortimer SA, Bai Y, Doudna JA (2011) Crystal structure of the HCV IRES central domain reveals strategy for start-codon positioning. Structure 19: 1456–1466. 22000514 10.1016/j.str.2011.08.002 PMC3209822

[8] Sasaki J, Nakashima N (2000) Methionine-independent initiation of translation in the capsid protein of an insect RNA virus. Proc Natl Acad Sci U S A 97: 1512–1515. 10660678 10.1073/pnas.010426997 PMC26465

[9] Sasaki J, Nakashima N (1999) Translation initiation at the CUU codon is mediated by the internal ribosome entry site of an insect picorna-like virus in vitro. J Virol 73: 1219–1226. 9882324 10.1128/jvi.73.2.1219-1226.1999 PMC103943

[10] Jan E, Sarnow P (2002) Factorless ribosome assembly on the internal ribosome entry site of cricket paralysis virus. J Mol Biol 324: 889–902. 12470947 10.1016/s0022-2836(02)01099-9

[11] Wilson JE, Pestova TV, Hellen CU, Sarnow P (2000) Initiation of protein synthesis from the A site of the ribosome. Cell 102: 511–520. 10966112 10.1016/s0092-8674(00)00055-6

[12] Pestova TV, Hellen CU (2003) Translation elongation after assembly of ribosomes on the Cricket paralysis virus internal ribosomal entry site without initiation factors or initiator tRNA. Genes Dev 17: 181–186. 12533507 10.1101/gad.1040803 PMC195975

[13] Jan E, Kinzy TG, Sarnow P (2003) Divergent tRNA-like element supports initiation, elongation, and termination of protein biosynthesis. Proc Natl Acad Sci U S A 100: 15410–15415. 14673072 10.1073/pnas.2535183100 PMC307581

[14] Yamamoto H, Nakashima N, Ikeda Y, Uchiumi T (2007) Binding mode of the first aminoacyl-tRNA in translation initiation mediated by Plautia stali intestine virus internal ribosome entry site. J Biol Chem 282: 7770–7776. 17209036 10.1074/jbc.M610887200

[15] Nakashima N, Uchiumi T (2008) Functional analysis of structural motifs in dicistroviruses. Virus Res 139: 137–147. 18621089 10.1016/j.virusres.2008.06.006

[16] Jan E (2006) Divergent IRES elements in invertebrates. Virus Res 119: 16–28. 16307820 10.1016/j.virusres.2005.10.011

[17] Nishiyama T, Yamamoto H, Shibuya N, Hatakeyama Y, Hachimori A, et al (2003) Structural elements in the internal ribosome entry site of Plautia stali intestine virus responsible for binding with ribosomes. Nucleic Acids Res 31: 2434–2442. 12711689 10.1093/nar/gkg336 PMC154222

[18] Costantino D, Kieft JS (2005) A preformed compact ribosome-binding domain in the cricket paralysis-like virus IRES RNAs. RNA 11: 332–343. 15701733 10.1261/rna.7184705 PMC1370722

[19] Schuler M, Connell SR, Lescoute A, Giesebrecht J, Dabrowski M, et al (2006) Structure of the ribosome-bound cricket paralysis virus IRES RNA. Nat Struct Mol Biol 13: 1092. 17115051 10.1038/nsmb1177

[20] Pfingsten JS, Costantino D, Kieft JS (2006) Structural Basis for Ribosome Recruitment and Manipulation by a Viral IRES RNA. Science 314: 1450. 17124290 10.1126/science.1133281 PMC2669756

[21] Pfingsten JS, Castile AE, Kieft JS (2010) Mechanistic Role of Structurally Dynamic Regions in Dicistroviridae IGR IRES. J Mol Biol 395: 205–217. 19878683 10.1016/j.jmb.2009.10.047 PMC2788014

[22] Nishiyama T, Yamamoto H, Uchiumi T, Nakashima N (2007) Eukaryotic ribosomal protein RPS25 interacts with the conserved loop region in a dicistroviral intergenic internal ribosome entry site. Nucleic Acids Res 35: 1514–1521. 17287295 10.1093/nar/gkl1121 PMC1865070

[23] Landry DM, Hertz MI, Thompson SR (2009) RPS25 is essential for translation initiation by the Dicistroviridae and hepatitis C viral IRESs. Genes Dev 23: 2753–2764. 19952110 10.1101/gad.1832209 PMC2788332

[24] Jang CJ, Lo MC, Jan E (2009) Conserved element of the dicistrovirus IGR IRES that mimics an E-site tRNA/ribosome interaction mediates multiple functions. J Mol Biol 387: 42–58. 19361441 10.1016/j.jmb.2009.01.042

[25] Costantino DA, Pfingsten JS, Rambo RP, Kieft JS (2008) tRNA-mRNA mimicry drives translation initiation from a viral IRES. Nat Struct Mol Biol 15: 57–64. 18157151 10.1038/nsmb1351 PMC2748805

[26] Pfingsten JS, Costantino DA, Kieft JS (2007) Conservation and diversity among the three-dimensional folds of the Dicistroviridae intergenic region IRESes. J Mol Biol 370: 856–869. 17544444 10.1016/j.jmb.2007.04.076 PMC1974883

[27] Chang YN, Pirtle IL, Pirtle RM (1986) Nucleotide sequence and transcription of a human tRNA gene cluster with four genes. Gene 48: 165–174. 3557125 10.1016/0378-1119(86)90362-8

[28] Zhu J, Korostelev A, Costantino DA, Donohue JP, Noller HF, et al (2011) Crystal structures of complexes containing domains from two viral internal ribosome entry site (IRES) RNAs bound to the 70S ribosome. Proc Natl Acad Sci U S A 108: 1839–1844. 21245352 10.1073/pnas.1018582108 PMC3033271

[29] Hertz MI, Thompson SR (2011) In vivo functional analysis of the Dicistroviridae intergenic region internal ribosome entry sites. Nucleic Acids Res 39: 7276–7288. 21646337 10.1093/nar/gkr427 PMC3167618

[30] Carrasco L, Battaner E, Vazquez D (1974) The elongation steps in protein synthesis by eukaryotic ribosomes: effects of antibiotics. Methods Enzymol 30: 282–289. 4605255 10.1016/0076-6879(74)30031-6

[31] Szer W, Kurylo-Borowska Z (1970) Effect of edeine on aminoacyl-tRNA binding to ribosomes and its relationship to ribosomal binding sites. Biochim Biophys Acta 224: 477–486. 5498079 10.1016/0005-2787(70)90580-0

[32] Wilson JE, Powell MJ, Hoover SE, Sarnow P (2000) Naturally occurring dicistronic cricket paralysis virus RNA is regulated by two internal ribosome entry sites. Mol Cell Biol 20: 4990–4999. 10866656 10.1128/mcb.20.14.4990-4999.2000 PMC85949

[33] Kanamori Y, Nakashima N (2001) A tertiary structure model of the internal ribosome entry site (IRES) for methionine-independent initiation of translation. Rna 7: 266–274. 11233983 10.1017/s1355838201001741 PMC1370084

[34] Ren Q, Wang QS, Firth AE, Chan MM, Gouw JW, et al (2012) Alternative reading frame selection mediated by a tRNA-like domain of an internal ribosome entry site. Proc Natl Acad Sci U S A 109: E630–639. 22247292 10.1073/pnas.1111303109 PMC3306683

[35] Jang CJ, Jan E (2010) Modular domains of the Dicistroviridae intergenic internal ribosome entry site. RNA 16: 1182–1195. 20423979 10.1261/rna.2044610 PMC2874170

[36] Dreher TW (2009) Role of tRNA-like structures in controlling plant virus replication. Virus Res 139: 217–229. 18638511 10.1016/j.virusres.2008.06.010 PMC2676847

[37] Matsuda D, Dreher TW (2004) The tRNA-like structure of Turnip yellow mosaic virus RNA is a 3'-translational enhancer. Virology 321: 36–46. 15033563 10.1016/j.virol.2003.10.023

